# Investigating the repurposing potential of immune checkpoint inhibition for cancer treatment using Mendelian randomisation

**DOI:** 10.21203/rs.3.rs-9656350/v1

**Published:** 2026-07-28

**Authors:** Tessa Bate, Mei Dong, Yuxi Liu, Mathias Seviiri, M. Catherine Brown, Joshua Atkins, Karl Smith-Byrne, The Practical Consortium, Rayjean Hung, Matthew Law, Mark Iles, Hadis Ghajari, Serigne Lo, Alexander Gusev, Brett Vanderwerff, Sebastian Zoellner, Geoffrey Liu, Wei Xu, Richard Martin, James Yarmolinsky, Philip Haycock, International Lung Cancer Consortium (ILCCO)

**Affiliations:** University of Bristol; Dalla Lana School of Public Health, University of Toronto; Brigham and Women’s Hospital; QIMR Berghofer Medical Research Institute; Princess Margaret Cancer Centre, University Health Network; University of Oxford; Cancer Epidemiology Unit, University of Oxford; The Institute of Cancer Research http://orcid.org/0000-0001-6316-5484; QIMR Berghofer; University of Leeds; QIMR Berghofer; Melanoma Institute Australia, University of Sydney; Dana-Farber Cancer Institute; University Health Network / Princess Margaret Cancer Centre; University Health Network, Toronto; University of Bristol; Imperial College of London; University of Bristol

## Abstract

Programmed cell death protein 1 (PD-1) and programmed death ligand-1 (PD-L1) are immune checkpoint proteins involved in tumour evasion of immune suppression. Inhibitors of these proteins are approved to treat cancer patients restricted by criteria such as site and immunologically ‘hot’ tumour features. To assess their repurposing potential to additional cancer patient populations, we used a Mendelian randomisation approach to investigate the effects of germline proxied plasma PD-1 or PD-L1 lowering on all-cause or cancer-specific mortality risk after diagnosis of cancers with (breast, colorectal, lung, melanoma) or without (ovarian, prostate) PD-1 and PD-L1 inhibitor approvals.

Across the four studied cancers with current PD-1/L1 inhibitor approvals, hazard ratios (HRs) for mortality [95% confidence intervals] per standard deviation decrease in germline proxied plasma protein levels were PD-1:0.91 [0.84–0.98]; PD-L1:0.98 [0.92–1.05]. This suggests failure of the PD-L1 instrument to recapitulate known pharmaceutical effects of PD-L1 inhibition, potentially due to germline proxies not robustly reflecting tumour PD-L1 mechanisms. There was evidence to support PD-1 inhibitor repurposing for a novel indication, ovarian cancer treatment (HR = 0.88 [0.78–0.99]), and to broader colorectal (HR = 0.84 [0.71–1.00]) and early-stage lung (HR = 0.75 [0.61–0.93]) cancer populations than current approvals. Where possible, methodological considerations were addressed but findings should be interpreted with caution.

## Introduction

In 2022, there were an estimated 20 million new cancer cases and 9.7 million deaths attributed to cancer globally [1]. As the global population size and proportion of older individuals increases, cancer incidence and mortality are expected to continue to increase [1], and additional cancer treatment strategies are required to address the gap in current treatment options [2]. Drug repurposing, the use of an approved drug with established safety profiles for an indication different to its original indication [3, 4], offers a potential strategy for the identification of novel treatments for cancer patients.

One possible source of repurposing opportunities for cancer treatment are approved medications that target cancer hallmarks [5], the features of cancer cells which are common across anatomical sites [6]. Binding of the immune checkpoint proteins programmed cell death protein 1 (PD-1) and its ligand programmed death ligand-1 (PD-L1) triggers a signalling cascade which results in inhibition of T cell activation through dephosphorylation of T cell antigen receptors [7, 8]. PD-L1 expression can be upregulated on tumour cells, thus inhibiting the interaction between PD-1 and PD-L1 can restore anti-cancer immune responses through T cell activation [8–12]. Inhibitors of PD-1 and PD-L1 thus target the cancer hallmark of evading immune destruction [5, 6] and are currently approved in the United Kingdom by the Medicines and Healthcare products Regulatory Agency (MHRA) for over 80 indications across more than 15 anatomical sites (e.g. breast, cervix, colorectum, endometrium, lung, melanoma, urothelial, etc) (Supplementary Table 1), as well as site-agnostic cancer indications [13–24]. In many cases, approvals of PD-1 and PD-L1 inhibitors are for highly restricted cancer patient populations. For example, approvals may be restricted by clinical features such as advanced stage or treatment history, or with respect to features of immunologically ‘hot’ (i.e. ‘immune-inflamed’) tumours such as tumour PD-L1 expression and tumour mutational burden (TMB) [13–25]. Prior studies have observed associations between decreased pre-treatment PD-1 and PD-L1 sera levels and improved survival or response to immune checkpoint inhibitors for cancer patients, although such studies have only included small numbers of participants and did not always have strong evidence to support these associations [26–29].

Existing genetic data from large-scale genome-wide association studies (GWAS) enable causal inference through Mendelian randomisation (MR), providing an opportunity to evaluate the repurposing potential of medications without additional data collection or patient burden [30]. MR uses germline genetic variants as instruments (“proxies”) to evaluate causal relationships between exposures and disease outcomes [3], leveraging the random allocation of germline genetic variants at meiosis to reduce vulnerability to confounding and reverse causation bias [3, 31–33]. In drug-target MR, the effects of a drug are genetically proxied as the exposure of interest. When using cancer survival outcomes in drug-target MR, there are considerations such as validity of instruments in the tertiary prevention setting or in proxying tumour mechanisms, and challenges such as limited statistical power and potential index event bias (also known as collider bias) [30, 31, 34–37]. Consequently, the application of these methods to cancer tertiary prevention settings also warrants careful evaluation.

We therefore investigated the repurposing potential of PD-1 or PD-L1 inhibitors in cancer treatment, using drug-target MR to assess the effects of genetically proxied lowering of PD-1 or PD-L1 plasma protein levels on risk of either all-cause or cancer-specific mortality for participants diagnosed with breast, colorectal, lung, melanoma, ovarian or prostate cancer. PD-1 or PD-L1 inhibitors are currently approved for treatment of breast, colorectal, lung cancer and melanoma, thus for these sites we investigated repurposing to less restricted patient populations than current indications, while for ovarian and prostate cancer we investigated repurposing of these medications to anatomical sites with no current indications. We also comprehensively evaluated the sensitivity of results to violations of MR assumptions.

## Results

### Instrument construction

Individual genetic instruments in PD-1 and PD-L1 instrument sets had F-statistics between 21.5 and 1304.3 or 21.1 and 2371.8, respectively (Supplementary Table 2–3). When accounting for LD between instruments in these sets, the entire PD-1 and PD-L1 instrument sets had F-statistics between 25.7 and 40.5 or 42.0 and 45.0, respectively (Supplementary Table 4). Therefore, as instruments with F-statistics greater than 10 are considered to be strong, there was low risk of weak instrument bias in our analyses. The lead PD-1 instrument (rs75960776) explained 2.48% of the variance in plasma PD-1 protein levels, while the lead PD-L1 instrument (rs822341) explained 4.47% of the variance in plasma PD-L1 protein levels (Supplementary Table 2–3).

### Power estimates

We estimated hazard ratios (HRs) detectable at 80% power per standard deviation (SD) change in PD-1 or PD-L1 plasma protein levels, assuming a two-sided alpha of 5% and given the number of participants and mortality events for each cancer survival GWAS [38, 39]. There was estimated 80% power to detect HRs ranging from 0.60 to 0.83 across cancer sites for the lead PD-1 instrument (rs75960776) and from 0.65 to 0.86 for the lead PD-L1 instrument (rs822341) (Supplementary Table 5). This suggests that we may have limited power to detect small or modest effect sizes, particularly for effects on melanoma and ovarian cancer survival. We used an additional approach to assess reliability of these power estimates and found general concordance between these methods, with 74.1% to 82.7% power estimated to detect the HRs obtained using the first approach (Supplementary Table 6, further described in Methods).

### Summary-level MR estimator

Pharmaceutical inhibition of both PD-1 and PD-L1 is protective for cancer-specific and all-cause mortality. When findings across sites with current PD-1/L1 inhibitor approvals (breast, colorectum, lung, melanoma) were combined in a random effects meta-analysis, there was evidence to support an effect of PD-1 (HR per SD decrease in genetically proxied PD-1 level [95% CI] = 0.91 [0.84, 0.98], Q = 1.70, heterogeneity p-value = 0.64), but not PD-L1 (HR per SD decrease in genetically proxied PD-L1 level [95% CI] = 0.98 [0.92, 1.05], Q = 5.16, heterogeneity p-value = 0.16) lowering on decreased risk of mortality.

**Figure 1:**
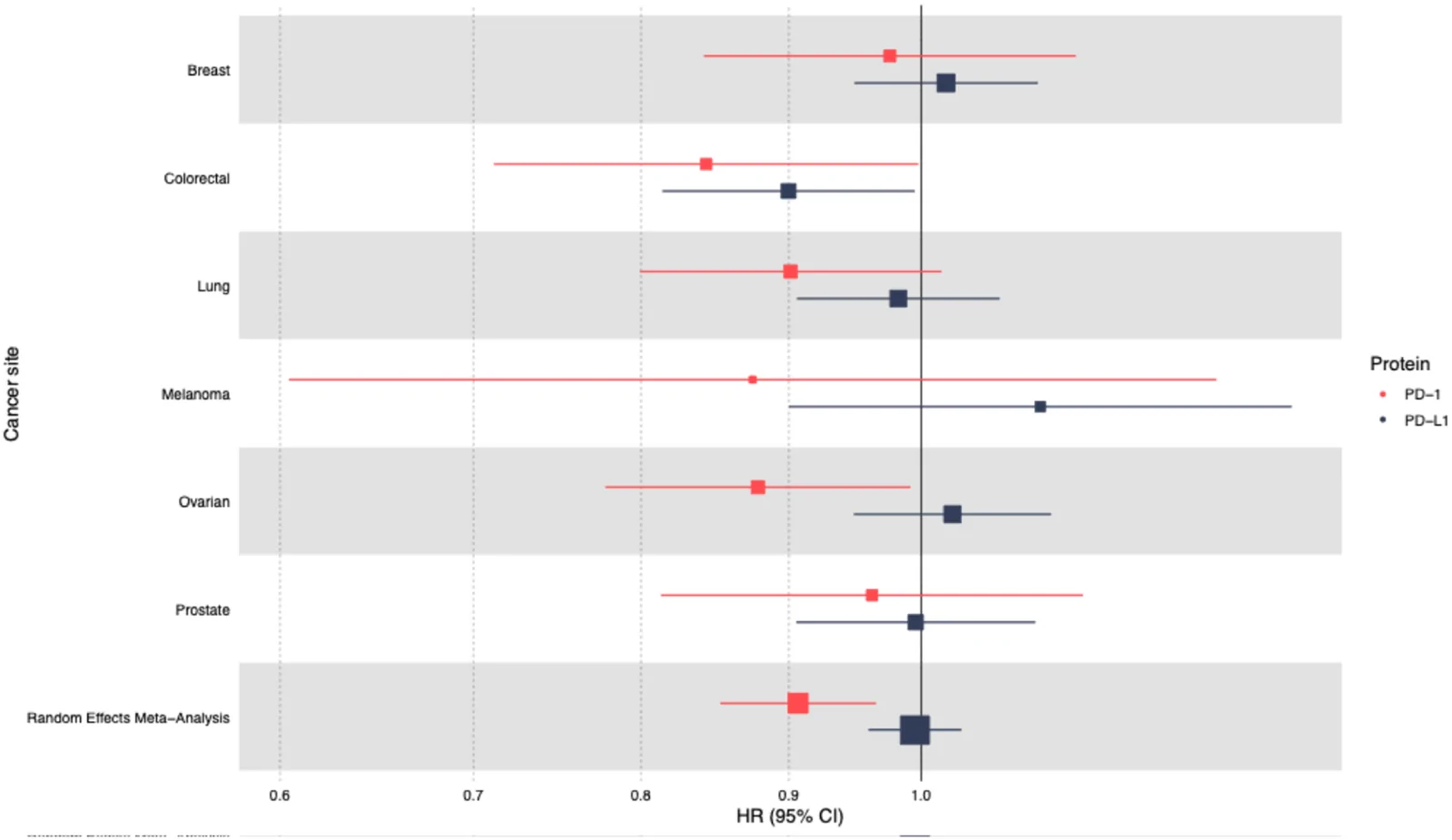
Hazard ratios (95% confidence intervals) for risk of either cancer-specific (breast, colorectal, melanoma, prostate cancer) or all-cause (lung, ovarian cancer) mortality per standard deviation (SD) decrease in genetically proxied PD-1 or PD-L1 plasma protein level for breast, colorectal, lung, melanoma, ovarian or prostate cancer patients.

Across all included cancer sites, effect estimates for genetically proxied plasma PD-1 protein level were consistent with the protective effect of pharmaceutical PD-1 inhibition (Fig. 1). However, wide 95% confidence intervals (CIs) for estimates aside from colorectal and ovarian cancer sites were also compatible with small increased risk of mortality (Fig. 1). Per SD decrease in genetically proxied PD-1 protein levels, HRs for cancer-specific mortality [95% CI] were: 0.98 [0.84, 1.13] for breast, 0.84 [0.71, 1.00] for colorectal, 0.87 [0.60, 1.27] for melanoma and 0.96 [0.81, 1.14] for prostate cancer patients (Fig. 1). Per SD decrease in genetically proxied PD-1 protein levels, HRs for all-cause mortality were: 0.90 [0.80, 1.02] for lung and 0.88 [0.78, 0.99] for ovarian cancer patients (Fig. 1). In a random-effects meta-analysis, we found that the overall effect for PD-1 on mortality combined across the six cancer sites was: HR = 0.91 [0.85, 0.96] (Q = 2.44, heterogeneity p-value = 0.79) (Fig. 1).

In contrast, genetically proxied PD-L1 inhibition was not consistently associated with cancer-specific or all-cause mortality (Fig. 1). Per SD decrease in genetically proxied PD-L1 plasma protein levels, HRs for cancer-specific mortality were: 1.02 [0.95, 1.10] for breast, 0.90 [0.81, 0.99] for colorectal, 1.10 [0.90, 1.34] for melanoma and 1.00 [0.91, 1.10] for prostate cancer patients (Fig. 1). Per SD decrease in genetically proxied PD-L1 plasma protein levels, HRs for all-cause mortality were: 0.98 [0.91, 1.06] for lung and 1.03 [0.95, 1.11] for ovarian cancer patients (Fig. 1). In a random-effects meta-analysis, we found that the overall effect for PD-L1 on mortality combined across the six cancer sites was: HR = 1.00 [0.96, 1.03] (Q = 5.91, heterogeneity p-value = 0.32) (Fig. 1). There was evidence to support a difference in the meta-analysed effects of PD-1 compared to PD-L1 protein lowering on risk of mortality combined across the six sites (Z=−2.53, p = 0.01).

No clear visual outliers were observed for these findings with respect to effects of individual variants in the instrument sets on cancer survival (Supplementary Fig. 1–2). There were also no clear visual outliers for instrument effects on protein levels and cancer survival compared to other variants in instrument sets (Supplementary Fig. 3–4). Across all included MR analyses, there was some visual indication of asymmetry of funnel plots, particularly for PD-1 instruments (Supplementary Fig. 5–6). Although this may suggest potential violation of the exclusion restriction MR assumption [40], asymmetry could also reflect the inclusion of correlated variants within the instrument sets.

### Colocalisation analyses

**Figure 2:**
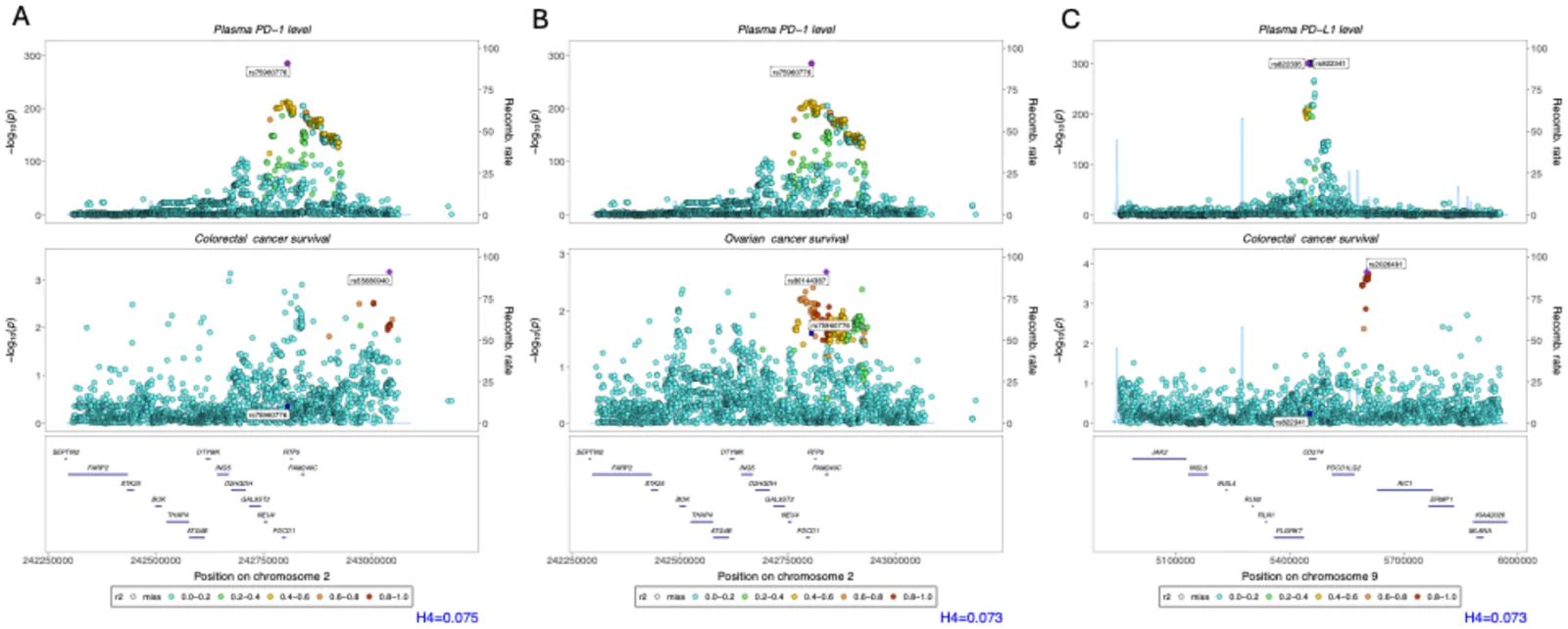
Regional association plots of protein-cancer survival effects with MR evidence (p<0.05), with LD between variants in genomic regions estimated based on reference panel of 10,000 UK Biobank participants of European ancestry. H4=probability of shared causal variant for protein level and cancer survival traits.

For the findings with nominal evidence of association in MR analyses (p < 0.05) (Fig. 1), there was little evidence of colocalisation between genetic signals for protein levels and cancer survival [41] (Supplementary Table 7, Fig. 2). As a sensitivity analysis, given the anticipated low power of colocalisation analyses, we also assessed linkage disequilibrium (LD) between the lead variants for PD-1 or PD-L1 protein levels and the lead variants for cancer survival in the relevant protein-coding genomic regions, rather than across the entire genomic regions as in colocalisation analyses. The lead PD-1 and PD-L1 genetic variants were not in strong LD with lead variants for cancer survival within these regions (LD R^2^ between lead variants range=[2×10^− 4^, 0.38]), also suggesting low probability of these traits sharing the same causal variant (Supplementary Table 7). However, these results may also have been impacted by limited statistical power of the cancer survival GWAS.

### Collider bias

**Figure 3:**
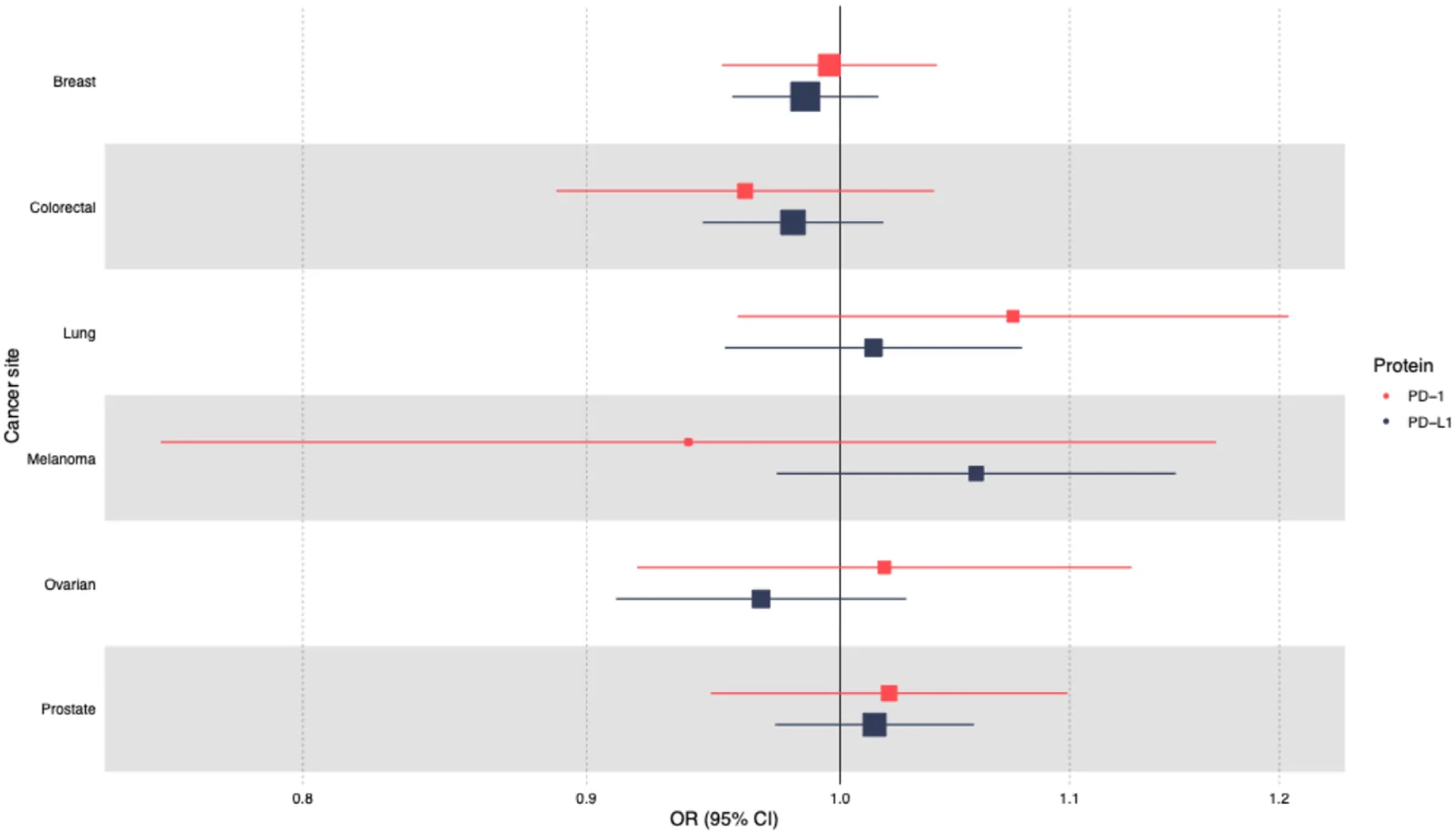
Odds ratios (95% confidence intervals) for risk of cancer incidence per standard deviation (SD) decrease in genetically proxied PD-1 or PD-L1 plasma protein level for breast, colorectal, lung, melanoma, ovarian or prostate cancer patients.

There was a potential risk of collider bias affecting these analyses, as participants were selected into the outcome studies based on disease diagnosis [42]. This could result in induction of spurious associations between exposures and risk of mortality outcomes, or attenuation of exposure-outcome effects [43]. Therefore, the risk of collider bias was assessed by investigating whether the PD-1 or PD-L1 genetically proxied exposures were associated with incidence of cancer at each included site. We found little evidence for associations with cancer risk (Fig. 3). Per SD decrease in genetically proxied PD-1 protein levels, odds ratios (ORs) were: 1.00 [0.95, 1.04] for breast cancer, 0.96 [0.89, 1.04] for colorectal cancer, 1.07 [0.96, 1.20] for lung cancer, 0.94 [0.75, 1.17] for melanoma, 1.02 [0.92, 1.13] for ovarian cancer and 1.02 [0.95, 1.10] for prostate cancer (Fig. 3). Per SD decrease in genetically proxied PD-L1 protein levels, ORs were: 0.99 [0.96, 1.02] for breast cancer, 0.98 [0.94, 1.02] for colorectal cancer, 1.01 [0.95, 1.08] for lung cancer, 1.06 [0.97, 1.15] for melanoma, 0.97 [0.91, 1.03] for ovarian cancer and 1.01 [0.97, 1.06] for prostate cancer (Fig. 3). Consequently, we interpret these results as indicating low risk of collider bias in our survival analyses and so the survival estimates (Fig. 1) were not adjusted for collider bias.

### Sensitivity analyses

We constructed instruments to proxy PD-1 and PD-L1 protein levels based on plasma in a population not restricted to cancer patients. As the MR relevance assumption can be violated due to differences between populations used for exposure and outcome measures, we investigated whether our lead PD-1 and PD-L1 instruments were associated with respective protein levels in cancer patient populations. We also investigated whether these instruments were associated with expression of respective coding genes in tissues relevant to the mechanism of action of these medications (PD-1: T cell *PDCD1* expression; PD-L1: tumour cell *CD274* expression). Although there was evidence to support associations of these instruments with blood PD-1 or PD-L1 levels in cancer, including treated cancer, patient populations (Supplementary Results, Supplementary Fig. 7–8, Supplementary Table 8), there was no strong evidence to support their associations with protein-coding genes in biologically relevant tissues (Supplementary Results, Supplementary Fig. 9–11, Supplementary Table 9–10).

Our findings were largely consistent with increasing stringency of instrument construction criteria and across instrument sets in which individual instruments had been iteratively excluded from analyses (Supplementary Results, Supplementary Fig. 12–14). A phenome-wide association study (PheWAS) conducted to assess risk of horizontal pleiotropy in our analyses identified phenotypes with potential pleiotropic effects (Supplementary Results, Supplementary Table 11). For example, the lead PD-L1 instrument was associated with risk of ulcerative colitis (OR = 1.11, FDR p-value = 5.6×10^− 5^) and inflammatory bowel disease (OR = 1.07, FDR p-value = 0.01) (Supplementary Results, Supplementary Table 11) which may be vertically pleiotropic as immune-related colitis is an adverse event of PD-L1 inhibitors [44]. However, whether the phenotypes observed in the PheWAS were likely vertically or horizontally pleiotropic was not always clear.

### Sub-group analyses

Where data permitted, sub-group analyses were performed based on patient and tumour characteristics (e.g. stage, treatment history, tumour molecular profiles). In our previously published protocol, we hypothesised that genetic effects would be stronger among patients diagnosed with early-stage compared with late-stage disease [45]. We did not specify *a priori* hypotheses for effect modification by other tumour and patient characteristics; therefore, findings from these exploratory sub-group analyses should be interpreted with caution.

### Breast cancer

There was very little evidence to support effects of lower genetically proxied plasma PD-1 or PD-L1 levels on breast cancer survival for the overall population (Fig. 1). In contrast, there was nominal evidence to support a potentially increased risk of mortality with PD-1 lowering for patients with oestrogen receptor (ER)+ progesterone receptor (PR)+ human epidermal growth factor receptor 2 (HER2)- tumours who had previously received chemotherapy (HR = 1.58 [1.03, 2.44]) and with PD-L1 lowering for patients with ER- PR- HER2 + tumours (HR = 1.47 [1.03, 2.11]) (Supplementary Fig. 16).

### Colorectal cancer

The MR estimates were consistent across colorectal cancer sub-groups, other than a stronger but still imprecise estimate for PD-L1 lowering on risk of mortality for stage 1 colorectal cancer patients compared to other stages (HR = 0.62 [0.37, 1.02] for stage 1, 0.94 [0.79, 1.13] for stages 2 and 3, 0.87 [0.73, 1.03] for stage 4) (Supplementary Fig. 17).

### Lung cancer

There was no strong evidence for an effect of PD-1 and PD-L1 lowering on lung cancer mortality when all stage patients were included in analyses (Fig. 1). However, when analyses were stratified by stage, there was stronger evidence to support effects of genetically proxied PD-1 and PD-L1 protein lowering on reduced risk of mortality in early-stage (stage I-II) lung cancer: HR = 0.75 [0.61, 0.93] per SD decrease in PD-1; 0.85 [0.73, 0.98] per SD decrease in PD-L1 (Supplementary Fig. 18). By contrast, the results for late-stage (stage III-IV) lung cancer were: HR = 0.99 [0.85, 1.16] per SD decrease in PD-1; 1.05 [0.94, 1.17] per SD decrease in PD-L1 (Supplementary Fig. 18).

## Discussion

This study has used drug-target MR to evaluate the repurposing potential of PD-1 and PD-L1 inhibitors for treatment of cancers at six anatomical sites. For four of these sites, breast, colorectal, lung cancer, and melanoma, PD-1 or PD-L1 inhibitors are currently approved for restricted patient populations, whereas there are no such approvals for patients diagnosed with ovarian or prostate cancer. When findings were combined across sites with current PD-1/L1 inhibitor approvals, there was evidence to support effects of our constructed PD-1 instrument on risk of mortality in a direction consistent with pharmaceutical inhibition. However, this was not the case for the PD-L1 instrument, suggesting potential failure of this instrument to recapitulate pharmaceutical PD-L1 inhibition. We observed some evidence to support a survival benefit of the genetically proxied effects of PD-1 inhibition for cancers at three anatomical sites (ovary, colorectum, early-stage lung), including one site without current PD-1 inhibitor approvals (ovary). However, despite strong *a priori* expectations for protective effects of these medications on cancer survival based on underlying mechanisms and current indications, the majority of findings did not support PD-1 inhibitor repurposing to cancer sites beyond their current indications. Our site-specific effects for PD-1 were likely impacted by low statistical power, as there was evidence to support effects of proxied PD-1 on cancer survival in meta-analyses. These findings for PD-1 should therefore be interpreted with caution due to low statistical power and findings for PD-L1 should be interpreted with strong caution due to potential lack of instrument validity to proxy biologically relevant tissue expression of PD-L1 for the medication mechanism of action.

We observed some evidence to support a protective effect of PD-1 protein lowering for ovarian cancer patients. There are currently no PD-1 inhibitors approved by the MHRA for site-specific ovarian cancer indications [15–18, 22] as there is a lack of robust clinical trial support for PD-1 inhibitor efficacy in ovarian cancer treatment [46–48]. A meta-analysis of clinical trials investigating the efficacy of monotherapy PD-1 or PD-L1 inhibition for treatment of recurrent or refractory ovarian cancer patients observed no evidence for a protective effect of these medications, as median overall survival achieved (median overall survival = 10.70 [9.23, 12.17] months) was shorter than previously reported for advanced or recurrent epithelial ovarian cancer patients (median overall survival = 14.6 months) [49]. This lack of immune checkpoint inhibitor efficacy for both PD-1 and PD-L1 inhibitors in ovarian cancer treatment has been proposed to be attributed to the immunologically ‘cold’ status and low PD-L1 expression of a large proportion of ovarian tumours [46–51]. One strategy suggested to improve immune checkpoint inhibitor efficacy for ‘cold’ tumours is combination with chemotherapy to convert ‘cold’ tumours to ‘hot’ tumours [50]. A meta-analysis of clinical trials investigating the efficacy of PD-1 or PD-L1 inhibitors in combination with chemotherapy for ovarian cancer treatment also did not observe strong evidence to support a survival benefit with this treatment combination (HR for overall survival for PD-1 or PD-L1 inhibitors with chemotherapy compared to chemotherapy alone = 0.85 [0.64, 1.14] [51]), although this estimate is in the same direction of effect as our MR estimate.

Consequently, our MR findings generally contrast with findings from prior studies, clinical trials and current drug indications. One potential explanation for this is due to low statistical power of the clinical trial meta-analyses or methodological differences between MR studies and randomised controlled trials (RCTs). For example, compared to our MR study which evaluated the effect of long-term PD-1 inhibition, clinical trials will evaluate the effect of PD-1 inhibition through pharmacological perturbation over shorter periods of time which may not be sufficient to lead to a survival benefit [31, 52]. Additionally, differences in population characteristics between clinical trials included in these meta-analyses and the ovarian cancer survival GWAS may explain these contradictory findings. The ovarian cancer survival GWAS used in our MR analyses included epithelial ovarian cancer patients who had previously been treated with cytoreductive surgery and chemotherapy [53], while the meta-analyses inclusion criteria were not restricted with respect to treatment history [49–51]. However, we also cannot rule out that our MR result is a false positive finding.

We also found evidence to support a repurposing opportunity for PD-1 inhibitors in colorectal cancer treatment. In contrast to ovarian cancer, PD-1 inhibitors are currently approved to treat colorectal cancer patient groups. However, the current MHRA immune checkpoint inhibitor indications for colorectal cancer treatment are restricted to deficient DNA mismatch repair (dMMR)/microsatellite instability-high (MSI-H) metastatic or unresectable tumours [15, 16, 22]. In contrast, our findings for genetically proxied PD-1 protein lowering on colorectal cancer survival were not limited to any specific stage or subtype patient population.

A study of participants of the phase II Neoadjuvant Immune Checkpoint Inhibition and Novel IO Combinations in Early-stage Colon Cancer (NICHE) trial observed a 26% response rate for treatment-naïve stage I-III proficient DNA mismatch repair (pMMR) colon adenocarcinoma patients treated with a combination of a PD-1 and cytotoxic T-lymphocyte associated protein 4 (CTLA-4) inhibitors prior to surgery [54]. All participants were defined as TMB-low (maximum TMB = 8.8 mutations/Mb), suggesting that PD-1 inhibitors may have clinical benefit even in immunologically ‘cold’ colon tumours [54]. However, this was a small (N participants = 31) study of a relatively restricted cancer patient population and cannot directly be compared to our analyses which have considered PD-1 or PD-L1 inhibitors as monotherapy [54]. The colorectal cancer survival GWAS did not provide information regarding participant MMR/microsatellite status [55], but only a minority of colorectal cancer patients have been estimated to have dMMR/MSI-H tumours (~ 15–17% in early stage, ~ 4% in metastatic stage [56–58]), suggesting that most of the patients in our analysis had pMMR tumours. Our findings suggest that PD-1 inhibitors could have repurposing potential for treatment of colorectal cancer patients regardless of tumour MMR/microsatellite status, which is somewhat supported by the findings of the study of NICHE trial participants [54]. However, this relies on the assumption that patients with dMMR/MSI-H colorectal tumours were not overrepresented in our analyses, or that our findings were not driven by strong effects in patients with dMMR/MSI-H tumours only. Analyses in populations of colorectal cancer patients stratified by MMR/microsatellite status are thus required to further assess efficacy of PD-1 and PD-L1 inhibitors for colorectal cancer patients with pMMR/microsatellite stable tumours.

Additionally, we observed evidence to support repurposing of PD-1 inhibitors for early-stage lung cancer treatment. PD-1 and PD-L1 inhibitors are both currently approved by the MHRA for lung cancer treatment, although these approvals are for highly specific patient groups typically defined using criteria such as tumour PD-L1 and gene mutations (e.g. lack or presence of *EGFR, ALK, ROS1* mutations) (Supplementary Table 1) [13–24]. These current approvals however are not restricted to early- or late-stage lung cancer patients (Supplementary Table 1) [13–24]. There are current approvals of PD-1 and PD-L1 inhibitors for patients with resectable lung tumours (Supplementary Table 1) [13–24], which stage I-II (i.e. early-stage) tumours are generally considered to be [59]. There are also approvals of PD-1 and PD-L1 inhibitors for patients with locally advanced or metastatic (i.e. late-stage) lung tumours (Supplementary Table 1) [13–24].

Therefore, we observed an effect of genetically proxied PD-1 lowering consistent with pharmaceutical inhibition for early- but not late-stage lung cancer. This may be explained by MR study design as we have only considered PD-1 or PD-L1 protein lowering alone, while approvals are often part of complex treatment regimens, for example as neoadjuvant or adjuvant treatment and as part of combination therapies (e.g. alongside chemotherapies or targeted therapies) (Supplementary Table 1) [13–24]. Additionally, our finding may be explained by a lack of statistical power attributed to lower heritability of cancer survival for late- compared to early-stage tumour patients (detailed further below and in Supplementary Note 2). Although this was a pre-specified analysis detailed in our protocol [45], this finding arises from sub-group analyses and so may be spurious as a result of multiple testing. Consequently, our findings suggest PD-1 inhibitors may have repurposing potential beyond current restrictive criteria for early-stage lung cancer patients however this needs to be interpreted with caution. As previously discussed for colorectal cancer, this interpretation assumes that participants with patient or tumour features which contribute to PD-1 or PD-L1 inhibitor efficacy were not overrepresented in the lung cancer survival GWAS, and that our observed effect did not occur due to a large effect for only such patients.

We considered three key aspects of MR applications in these analyses: risk of collider bias, statistical power and instrument validity. We did not observe strong evidence to support risk of collider bias influencing our results and even though our PD-1 and PD-L1 instruments were constructed based on protein levels obtained from a ‘general population’, we observed evidence to support application of these to cancer patient populations (further explored in Supplementary Note 1). However, analyses were limited by low statistical power (further explored in Supplementary Note 2) which may have limited detection of effects of these instruments on survival for cancers these medications are currently approved to treat (breast, colorectum, lung, melanoma) (further explored in Supplementary Note 1). When effects across all four sites with current PD-1 or PD-L1 inhibitors (breast, colorectum, lung, melanoma) were combined in a meta-analysis, there was evidence to support effects of genetically proxied PD-1 but not PD-L1 lowering on risk of mortality. This lack of strong evidence for effects on breast, colorectum, lung cancer and melanoma survival may also be explained by differences between the populations these medications are approved to treat and the populations recruited to the respective cancer survival GWAS (further explored in Supplementary Note 1). Additionally, there was lack of strong evidence to support utility of our instruments to proxy expression of respective genes in tissues relevant to the mechanism of action of these medications (further explored in Supplementary Note 1). This was particularly an issue for the PD-L1 instruments, likely because instruments were constructed based on plasma protein measures while the biologically relevant tissues for PD-L1 levels are tumour tissues.

Our analyses were strengthened by including numerous sensitivity analyses to assess validity of methods with respect to traditional MR limitations (e.g. robustness to different instrument construction criteria) as well as limitations from applying these methods to survival among cancer patients (e.g. collider bias). Sensitivity analyses were as comprehensive as possible given certain data access limitations and have illustrated complexities of applying drug-target MR to tertiary cancer prevention settings, although we acknowledge that these may not be generalisable beyond this studied immune checkpoint protein pathway. Through inclusion of, to the best of our knowledge, all available large-scale cancer survival GWAS, we have been able to investigate the repurposing potential of these medications to cancer sites which contribute to a large proportion of the estimated global cancer incidence (48.7% in 2022) and mortality (46.4% in 2022) [1] without additional patient burden. However, only genetic data from populations of European ancestry have been used in analyses so the generalisability of our findings to populations of non-European ancestry warrants further investigation. With time, the number and ancestral diversity of participants recruited to both protein expression GWAS and cancer survival GWAS is anticipated to increase. Thus, repetition of comparable analyses with updated datasets or as part of meta-analyses could be performed in the future to not only improve generalisability of these findings to populations of non-European ancestry but also increase the statistical power of analyses and more closely match positive control analyses to current drug indications.

When considering the clinical translation of these MR findings, the focus of interpretation should be on direction rather than magnitude of effect sizes due to differences between long-term decreased PD-1 or PD-L1 protein levels proxied in MR and short-term pharmaceutical PD-1 or PD-L1 inhibition achieved in RCTs [31]. This is further complicated as the GWAS used to construct PD-1 and PD-L1 instruments measured protein levels in normalised protein expression units (log2 SD scale) [60] which have unclear translation to pharmaceutical PD-1 or PD-L1 inhibition. MR methods are also only able to proxy the on-target effects of exposures and so these analyses may have not captured any clinically relevant off-target effects [61]. Furthermore, only the effects of PD-1 or PD-L1 plasma protein lowering have been proxied using MR, whereas pharmaceutical inhibition of these proteins is achieved by different drugs for the same protein target which may have different properties such as binding affinity [62, 63]. Consequently, repurposing opportunities highlighted in our analyses may have underestimated or overestimated clinical treatment effects, or not be consistent across all PD-1 or PD-L1 inhibitors in clinical translation.

In conclusion, there was some evidence to support the repurposing potential of PD-1 inhibitors for ovarian cancer treatment (a potential novel indication), early-stage lung cancers and all colorectal cancers, not just dMMR/MSI-H metastatic or unresectable tumours. These findings have limited supporting evidence from current RCTs, although this could be explained by differences between MR and RCT designs. Alternatively, these findings could be false positive findings, for example resulting from poor instrument validity and low statistical power. Overall, while we have identified three additional cancer patient populations which could have potential clinical benefit with PD-1 inhibitor treatment, these findings need to be followed up in pre-clinical models or RCTs before more robust conclusions can be drawn. In this study, we have also highlighted considerations of applying drug-MR methods to cancer tertiary prevention which we hope will help guide future research in this area.

## Methods

### Mendelian randomisation

MR uses germline genetic variants as instruments (“proxies”) for an exposure of interest (e.g. decreased plasma PD-1 or PD-L1 protein levels) to evaluate the causal effect of this exposure on an outcome of interest (e.g. risk of mortality) [64]. The three core MR assumptions are known as: relevance (i.e. the instruments should be associated with the exposure of interest), exchangeability (i.e. there should be no confounders of the instrument-outcome relationship) and exclusion restriction (i.e. the instruments should only influence the outcome of interest via the exposure of interest) [64].

### Instrument construction

#### Exposures

Two exposures were genetically proxied: decreased PD-1 or PD-L1 plasma protein levels to reflect PD-1 or PD-L1 inhibition. Genetic instruments were constructed to proxy these exposures using summary statistics obtained from a large protein expression GWAS conducted with UK Biobank participants of majority (estimated ~ 94%) European ancestry [60] (Table 1).

#### Data cleaning

To maximise the number of genetic variants retained in the final instrument sets, the protein expression GWAS summary statistics were first restricted to variants also present in the summary statistics of a large cancer survival GWAS for each respective cancer site (Table 1). Thus, six site-specific instrument sets were constructed for each protein level exposure. Multi-allelic variants were excluded, and variants were filtered to retain variants with MAF ≥ 1%.

#### Instrument construction criteria

In the primary analyses, lenient instrument construction criteria were applied. The protein expression GWAS summary statistics obtained from data cleaning were restricted to variants associated with PD-1 or PD-L1 plasma protein level (p-value<5×10^− 6^, based on an inverse z-score transformed equivalent to rank variants as some variants had extremely small p-values which otherwise may be ordered incorrectly in clumping). Variants were further restricted to *cis* variants, defined using a genomic window size of ± 500kb either side of the relevant protein-coding gene (PD-1: *PDCD1*, chr2:242792033–242801060; PD-L1: *CD274*, chr9:5450503–5470566 in hg19). These variants were clumped (LD R^2^ < 0.3 based on a reference panel of 10,000 UK Biobank participants of European ancestry, clumping window size of 1050kb) to construct the final instrument sets.

### Power estimates

#### Variance in trait explained by instruments

As the PD-1 and PD-L1 instrument sets included correlated (‘LD-dependent’) variants, the variance in plasma PD-1 or PD-L1 level explained by only the lead instruments were estimated. F-statistics for individual variants in the instrument sets were also estimated to assess instrument strength, although these will be inflated due to inclusion of correlated variants in the instrument sets. We also estimated F-statistics for entire instrument sets, accounting for LD between variants using a LD reference panel of 10,000 UK Biobank participants of European ancestry with the MendelianRandomization R package [65, 66].

#### Primary method used to estimate statistical power

HRs detectable at 80% power for the effects of the lead instruments on each cancer survival outcome were estimated using the survSNP R package survSNP.power.table function [67]. Power was estimated based on the reported sample size and death proportion (number of mortality events/total sample size) of the cancer survival GWAS (Table 1), reported risk allele frequency of the lead instrument and assuming an additive genetic model, a 5% alpha and five-year survival estimates for each cancer site obtained from the Cancer Research UK Cancer Statistics Data Hub [68, 69]. The estimated HRs were scaled to reflect a SD change in PD-1 or PD-L1 plasma protein levels.

#### Sensitivity method used to estimate statistical power

An alternative method, the powerSurvEpi R package powerEpiCont.default function [39], was used to estimate power to detect the HRs obtained using the primary power estimation method, with the following assumptions: variance in plasma PD-1 and PD-L1 plasma protein levels is 1, alpha of 5%, and no correlation between protein levels and other covariates. We scaled the reported sample sizes from each cancer GWAS by the proportion of variance in plasma protein levels explained by the respective lead instrument [70] (e.g. if the lead instrument was estimated to explain 1% of the variance in plasma protein level and the cancer survival GWAS reported a sample size of 10,000 cancer patients, sample size would have been scaled to 100 participants). This was performed to assess the consistency of the primary power estimate to a different power estimation method. Thus, estimating ~ 80% power in the sensitivity method provides us with greater confidence in the primary power estimation method results.

#### Outcome datasets

Mortality for patients diagnosed with cancers at six anatomical sites was selected as the outcome of interest based on availability of mortality GWAS summary statistics from large consortia performed in individuals of European ancestry diagnosed with breast [71], colorectal [55], lung (unpublished; early-stage: https://www.ebi.ac.uk/gwas/publications/40746155 [72]), melanoma [73], ovarian [53], and prostate (unpublished) cancer (Table 1). For the breast, colorectal, melanoma and prostate cancer risk of mortality GWAS, the mortality endpoint was cancer-specific mortality (Table 1) [55, 71, 73] (unpublished, early-stage: [72]). Whereas, for the lung and ovarian cancer risk of mortality GWAS, the mortality endpoint was all-cause mortality (Table 1) [53] (unpublished).

There is some potential overlap in participants between the protein expression GWAS and melanoma survival GWAS as both datasets included participants from the UK Biobank (Table 1). However, the extent of this overlap is not expected to be large as ~ 315 UK Biobank participants were identified as having both a melanoma diagnosis (based on International Classification of Diseases (ICD)-9 and ICD-10 codes) and PD-1 or PD-L1 plasma protein levels measured, while the melanoma survival GWAS included 5,220 UK Biobank participants diagnosed with melanoma [73].

#### Data harmonisation

The PD-1 and PD-L1 instruments were oriented so the minor allele was defined as the effect allele using a reference panel of 10,000 UK Biobank participants of European ancestry. Then, the instruments were harmonised with genetic data for each of the cancer survival outcomes using the conservative TwoSampleMR package harmonisation option which excludes palindromic SNPs if unable to assign the positive strand based on variant allele frequency [37, 74].

#### Summary-level MR estimator

LD matrices for variants in each harmonised dataset were constructed with PLINK v1.90 [75–77] using a random sample of 10,000 UK Biobank participants of European ancestry as a reference panel. The MendelianRandomization R package was then used to perform the random-effects inverse-variance weighted (IVW) method [65, 66], accounting for LD between variants within each instrument set using the generated LD matrices. As all instruments were selected from *cis* regions, we did not perform sensitivity analyses with pleiotropy robust MR methods such as MR-Egger as these methods require instruments from multiple independent genomic regions. The MR-IVW effect estimates were multiplied by −1 to reflect genetically proxied inhibition of PD-1 or PD-L1 (i.e. lowered plasma protein levels). Directions of effect and 95% CIs consistent with the known clinical effect of pharmaceutical PD-1 or PD-L1 inhibition and p-values < 0.05 were interpreted as indicating moderate evidence for association in the MR analyses. Effect estimates and 95% CIs inconsistent with the expected direction and with p-values > 0.05 were interpreted as indicating weaker evidence.

The TwoSampleMR [37, 74] and ggforestplot [78] R packages were then used to visualise these results. Following the calculation of Wald ratios for each variant in the instrument sets using the TwoSampleMR package [37, 74], forest plots were generated to visualise the MR-Wald estimates for each variant compared to the MR-IVW estimate for the overall instrument set. Scatter plots were also generated to demonstrate trends between variant-protein level and variant-cancer survival effect estimates across included instruments [37, 74]. Funnel plots were produced to present the variance of MR-IVW estimates across the instrument sets [37, 74].

Prostate cancer mortality GWAS summary statistics were available for both fixed- and random-effects meta-analyses. The fixed-effects meta-analysis estimates were used as the primary prostate cancer mortality measure, while analyses using the random-effects meta-analysis estimates were explored in sensitivity analyses (results presented in the Supplementary materials).

### Colocalisation analyses

#### Primary method for colocalisation analyses

Colocalisation analyses were performed for any main analysis finding with supporting MR evidence (p-value < 0.05) to assess whether there was evidence to support shared causal variants across protein levels and cancer mortality. For all variants within ± 500kb genomic windows surrounding the respective protein-encoding gene (MAF > 0.01, restricted to biallelic SNPs), colocalisation analyses were performed using Pair-Wise Conditional analysis and Colocalisation analysis (PWCoCo) with default parameters [41]. Effects were defined as having supporting colocalisation evidence where probability of the H4 hypothesis (i.e. hypothesis for shared causal variant(s) in the region associated with both protein level and cancer survival) > 80%.

#### Additional methods for colocalisation analyses

The cancer mortality GWAS were expected to have low statistical power which can limit applicability of PWCoCo methods so two additional methods were employed to assess evidence for colocalisation. Regional association plots were generated using the geni.plots R package fig_region_stack function (https://github.com/jrs95/geni.plots) for the same variants in the protein-encoding genomic regions as used in PWCoCo, using a reference panel of 10,000 UK Biobank participants of European ancestry to estimate LD structure. LD between the lead exposure and lead outcome variants were also estimated using the same reference panel of 10,000 UK Biobank participants of European ancestry.

#### Collider bias

The risk of collider bias affecting the survival outcome analyses was assessed by investigating the effect of genetically proxied decreased plasma PD-1 and PD-L1 levels on cancer incidence for each of the included site outcomes (Supplementary Table 12) [81–85] (unpublished) (OpenGWAS id:ieu-a-987). This was performed using the same summary-level MR methods as described above. In the event that the instruments were associated with cancer incidence (p<0.05), the extent of collider bias was planned to be assessed and adjusted for using the SlopeHunter R package (https://github.com/Osmahmoud/SlopeHunter) [42, 86].

#### Sensitivity analyses

##### Investigating the association of genetic instruments with plasma protein levels in individuals with cancer diagnoses

The PD-1 and PD-L1 genetic instrument sets were constructed based on plasma protein levels measured from a population comprised of randomly selected, consortium selected and COVID-19 imaging cohorts of UK Biobank participants [60]. Thus, to assess the validity of the PD-1 and PD-L1 instruments for individuals with a cancer diagnosis, the effect of the lead instruments on respective plasma protein levels in different cancer patient cohorts was performed in the UK Biobank dataset [87, 88]. A cohort of UK Biobank participants with at least one plasma measurement of PD-1 or PD-L1 protein levels was constructed. This cohort was further subdivided into cohorts of participants with at least one cancer diagnosis (the “any cancer patient population”), at least one cancer diagnosis across the six cancer sites of interest (the “pooled cancer patient population”) or cancer diagnosis for one of the six cancer sites of interest (six site-specific cancer patient populations) based on ICD-9 and ICD-10 diagnoses. Additionally, cohorts of “general population” participants were constructed, one unrestricted with respect to cancer diagnoses and one excluding participants with any ICD-9/10 cancer diagnosis or self-reported cancer occurrence for comparison.

The cohorts were further restricted to participants with matching self-reported (FID:31) and genetic sex (FID:22001), ‘White British’ genetic ethnic grouping (FID:22006), no sex chromosome aneuploidy (FID:22019) and without relatedness to other participants based on inclusion in principal component analysis (PCA) calculations (FID:22020) (modified script obtained from: https://github.com/dnanexus/UKB_RAP/blob/main/end_to_end_gwas_phewas/gwas-phenotype-samples-qc.ipynb). The cancer patient cohorts were also restricted to patients with malignant, primary tumours (FID:40011).

The cancer patient cohorts were additionally stratified with respect to time between blood sample collection used for the protein measurement (FID:3166) and first cancer diagnosis (FID:40005). Participants were defined as having incident cancers if blood sampling was performed prior to first cancer diagnosis, or prevalent cancers if blood sampling was performed after first cancer diagnosis. For the “any” or “pooled” cancer patient cohorts, additional incident (blood sampling performed within 1 year prior to cancer diagnosis) and prevalent (blood sampling performed within 1, 5 and 10 years after cancer diagnosis) sub-cohorts were generated.

Protein expression was regressed on the respective lead instrument, adjusting for a minimal set of covariates: age at assessment centre visit when blood sample collected (FID:21003), genetic principal components (PCs) 1–5 (FID:22009) and sex (FID:31) (where relevant). Analyses were also performed including time between blood sampling and first cancer diagnosis (in days) as an interaction term with the genetic variant. Finally, analyses were performed adjusting for a full set of covariates aiming to more closely match the covariates adjusted for in the protein expression GWAS used in instrument construction [60]: age at assessment centre visit when protein level measured (FID:21003), genetic PCs 1–20 (FID:22009), sex (FID:31) (where relevant), genotype measurement batch (FID:22000), geographic location of first assessment centre visit (FID:54) and duration between blood sampling collection and protein level measurement. Estimates obtained from cohorts of fewer than 100 participants were not reported.

##### Investigating the association of genetic instruments with immune cell population gene expression

DICE (https://dice-database.org/) investigated the association between genetic variants and gene expression in different immune cells obtained from peripheral blood from 91 general population participants [89]. The lead PD-1 instrument was looked up in this dataset for associations with *PDCD1* (Gene Symbol:ENSG00000188389) expression in the following T cell populations: CD4 memory T regulatory cells, naïve CD4 T cells, activated naïve CD4 T cells, naïve CD4 T regulatory cells, follicular helper CD4 T cells, CD4 T helper 1 cells, CD4 T helper 1/17 cells, CD4 T helper 17 cells, CD4 T helper 2 cells, naïve CD8 T cells, activated naïve CD8 T cells. The purpose of these analyses was to assess PD-1 instrument validity for the biologically relevant cell population, which are T cells [7].

##### Investigating the association of genetic instruments with tumour gene expression

We intended to assess the association between the lead PD-L1 instrument and tumour *CD274* gene expression using TCGA data, as tumour cell PD-L1 level is assumed to be biologically relevant for the mechanism of action of PD-L1 inhibitors [7]. However, this was not possible due to lack of full access to complete TCGA data. The PancanQTL website reports FDR p<0.05 associations between genetic variants and tumour gene expression for different TCGA studies (https://gong_lab.hzau.edu.cn/PancanQTL) [90], thus we used this resource to perform tumour gene expression validation analyses.

The lead PD-L1 instrument was looked up in PancanQTL for associations with *cis CD274* (PD-L1 encoding gene) expression for any of the TCGA studies corresponding to cancer sites included in our analyses (breast: breast invasive carcinoma (BRCA); colorectum: colon adenocarcinoma (COAD), rectum adenocarcinoma (READ); lung: lung adenocarcinoma (LUAD), lung squamous cell carcinoma (LUSC); melanoma: skin cutaneous melanoma (SKCM); ovary: ovarian serous cystadenocarcinoma (OV); prostate: prostate adenocarcinoma (PRAD)). LD proxies of the lead PD-L1 instrument were identified using the gwasvcf R package get_ld_proxies function [91] with default parameters (5000kb tag distance, 5000 number of tag SNPs, LD R^2^>0.6) and a reference panel of 10,000 UK Biobank participants of European ancestry. These LD proxies were also looked up in PancanQTL for associations with *cis CD274* expression for corresponding TCGA studies.

We also investigated whether any reported *cis* tumour *CD274* eQTLs for the TCGA studies of interest were associated with risk of mortality for patients with cancers diagnosed at the same anatomical site. This analysis provided insight into whether proxying PD-L1 inhibition using tumour gene expression, rather than plasma protein levels, could be a more valid instrument strategy in this application. To perform this, *cis CD274* eQTLs (FDR p<0.05) for BRCA, COAD, READ, LUAD, LUSC, SKCM, OV or PRAD TCGA studies were obtained from PancanQTL. Where possible, the eQTL with strongest evidence for association with tumour *CD274* for each site was then used in MR analyses with the same risk of mortality outcomes as for the primary analyses. If this was not possible (e.g. due to the variant being missing from the cancer survival GWAS or being palindromic preventing data harmonisation), the eQTL with the next smallest p-value for association with *CD274* gene expression was selected as an instrument. If this was also not possible, an LD proxy of an eQTL was identified using the same methods as detailed previously. Wald ratios obtained were multiplied by −1 to reflect decreased tumour *CD274* expression.

##### Investigating the association of genetic instruments with anatomical site tissue gene expression

We further assessed PD-L1 instrument validity by assessing associations with gene expression at relevant anatomical sites but for non-tumour samples. GTEx (https://www.gtexportal.org/home/) reports measures of variant-gene expression associations across different tissue sites collected from deceased donors recruited from a general population [92]. The effect of the lead PD-L1 instrument on expression of *CD274* (PD-L1 encoding gene) in tissues corresponding to each cancer site (breast: breast mammary tissue; colorectal: sigmoid colon, transverse colon; lung; melanoma: skin – sun exposed lower leg; ovary; prostate) was explored in the GTEx version 8 dataset.

##### Robustness to instrument construction criteria

While the main analysis instrument construction methods aimed to maximise the number of variants retained in instrument sets to increase statistical power, alternative methods using more stringent instrument construction criteria were employed as sensitivity analyses. These instrument sets were constructed using the same methods as previously described for the main analyses, but changing one of the following criteria: p-value thresholds for associations with plasma protein levels (p<5×10^−7^, 5×10^−8^), genomic window size restrictions on either side of the genes encoding the respective proteins (±250kb, 100kb), LD thresholds (R^2^<0.2, 0.1, 0.001).

##### Iterative “leave-one-out” analyses

To investigate whether any individual instruments in the instrument sets were strongly influencing observed MR effects, the main summary-level MR analyses were repeated excluding one instrument at a time and visualised in comparison to the estimate for the entire set in a forest plot.

##### White blood cell count

Interactions between PD-1 and PD-L1 have been hypothesised to promote downstream inhibition of lymphocyte, including T cell, proliferation in addition to T cell apoptosis through pathways such as cytokine production [93, 94]. Therefore, the effects of genetically proxied PD-1 or PD-L1 plasma protein levels on white blood cell count were assessed. These analyses were proposed in our original protocol to serve two purposes: (1) as positive control analyses (i.e. if PD-1 or PD-L1 instruments were associated with decreased white blood cell counts this would support their validity), (2) to assess whether there is evidence to support effects of PD-1 or PD-L1 plasma protein levels on cancer survival occurring through white blood cell counts [45].

A UK Biobank cohort of participants with white blood cell (leukocyte) count (FID:30000) was constructed, excluding participants with PD-1 or PD-L1 plasma protein level measurements as these participants are expected to have been included in the protein expression GWAS [60]. This cohort was divided into participants with or without any ICD-9 or ICD-10 cancer diagnoses. The cancer patient cohort was also stratified into participants with white blood cell count measured prior to or following first cancer diagnosis. The same quality control measures as for the previously described UK Biobank cohorts were used in cohort construction. Genetic risk scores to proxy PD-1 and PD-L1 protein levels were generated for participants of each cohort using the respective primary analysis instruments, with alleles oriented to reflect decreased protein levels. White blood cell count at recruitment was regressed on PD-1 or PD-L1 genetic risk score, adjusted for sex (FID:54), first five genetic PCs (FID:22009), age (FID:21003) and age^2^.

##### Phenome-wide association study

PheWAS were performed for the lead PD-1 and PD-L1 instruments using the ieugwasr R package phewas function [95], excluding already studied phenotype outcomes (PD-1 or PD-L1 protein levels, relevant cancer incidences). PheWAS effect sizes were oriented to match alleles reflecting decreased PD-1 or PD-L1 plasma protein levels and an FDR was applied to p-values to adjust for multiple testing.

### Sub-group analyses

Where data were available, summary-level MR was performed to investigate the effect of genetically proxied PD-1 or PD-L1 on survival for subgroup populations. Several different sub-groups were available for the breast cancer survival GWAS, including those based on age at diagnosis, treatment history and hormone receptor status [71]. Colorectal cancer survival GWAS data was available for sub-groups based on stage or subtype [55]. Lung cancer survival GWAS data was also available for early- or late-stage patient sub-groups (unpublished, [72]). Our pre-specific sub-group analyses were for treatment history and tumour stage sub-groups [45].

### Software and pre-registration

Analyses were performed using PLINKv1.9, PLINKv2.0 [76, 77, 96] and Rv4.3.3 with the reported R packages. This work was carried out using the computational facilities of the Advanced Computing Research Centre, University of Bristol (http://www.bristol.ac.uk/acrc/) or the UK Biobank Research Analysis Platform.

The planned methods for these analyses were previously described in the following protocol: [45]. One key change to analyses compared to the protocol is that the planned analyses for head and neck cancer survival outcomes were not performed due to lack of data availability.

## Supplementary Material

Supplementary Files

This is a list of supplementary files associated with this preprint. Click to download.

- supplementarymaterialsimmunecheckpointinhibitionmanuscript120526.docx
- supplementarytablesimmunecheckpointinhibitionmanuscript.xlsxs

## Figures and Tables

**Table 1:** Description of genome-wide association studies used in analyses.

Dataset	Phenotype	Reference	Consortia	Population	N participants	N mortality events	Covariates	Phenotype measurement
Exposures	Plasma PD-1 level	[60]	UK Biobank	Individuals of majority European ancestry with genotype and (Olink) protein expression data available	54,219 (combined discovery and replication cohorts)	-	Discovery • Age, age^2^ • Sex • Age × sex, age^2^ × sex • Batch • UK Biobank centre • UK Biobank genetic array • Time between blood sampling and measurement • Genetic PC1–20 Replication • All covariates as in discovery cohort • Participant preselection status	Olink protein expression measurements transformed to Normalized Protein eXpression (NPX) units.
	Plasma PD-L1 level							
Outcomes	Breast cancer survival	[71]	BCAC	Female breast cancer patients of European ancestry	91,686	7,531	None - primary analyses were unadjusted	Breast cancer-specific survival
	Colorectal cancer survival	[55]	ISACC	Individuals of European ancestry diagnosed with CRC	16,964	4,010	• Age at diagnosis • Sex • Genotyping platform and study • Genetic PC1–5	CRC-specific survival, defined as number of days from CRC diagnosis to death due to CRC causes or end of follow-up. Causes of death were defined using linkage to registries or active follow-up.
	Lung cancer survival	(Unpublished, early-stage: [72])	ILCCO, DFCI, GE, MGI	(Unpublished)	12,521	7,218	ILCCO • Age • Sex • Study site • Genetic PC1–5 DFCI • Age • Sex • Sequencing panel • Genetic PC1–10 GE [79] • Age • Sex • Genetic PC1–5 MGI • Age • Sex • Genetic PCs	All-cause mortality
	Melanoma survival	[73]	MIA, UK Biobank	Cutaneous melanoma patients of European ancestry	11,201	1,041	MIA • Age • Sex • Genetic PC1–10 • Genotyping batch UK Biobank • Age • Sex • Genetic PC1–10	Melanoma-specific mortality, defined by clinical records and linkage to registries
	Ovarian cancer survival	[53]	OCAC	Ovarian cancer patients of European ancestry who had previously received first line treatment (cytoreductive surgery)	2,901	1,656*	• Study • Genetic PC1 and PC2 • Binary indicator of residual disease • Stage • Histology • Grade • Age at diagnosis	Progression-free survival and overall survival
	Prostate cancer survival	(Unpublished)	PRACTICAL	(Unpublished)	67,758	7,914	• Genetic PCs • Age at diagnosis	Prostate cancer-specific mortality

For each genome-wide association study used to obtain genetic association data for the exposures and outcomes studies, the phenotype of interest, consortia, population characteristics and number (N) of participants these summary statistics were obtained from, number of mortality events observed, covariates which were accounted for, and how the outcome phenotype was measured.

Abbreviations: PD-1=programmed cell death protein-1, PD-L1=programmed cell death ligand-1, PCs=principal components, BCAC=breast cancer association consortium, ISACC=International Survival Analysis in Colorectal Cancer Consortium, CRC=colorectal cancer, DFCI=Dana Faber Cancer Institute, ILCCO=International Lung Cancer Consortium, MGI = Michigan Genomics Initiative, GE=Genomics England, MIA=Melanoma Institute Australia, OCAC=Ovarian Cancer Association Consortium, PRACTICAL=Prostate Cancer Association Group to Investigate Cancer Associated Alterations in the Genome.

* OCAC ovarian cancer survival GWAS number of mortality events was estimated based on the death proportion of 11 OCAC studies (AUS, BAV, BEL, HAW, HSK, MAC, MAL, MAY, NCO, NEC, PVD) (0.571)[80] and the ovarian cancer GWAS sample size (2901)[53].

## Data Availability

These analyses have exclusively used existing data which may be available from the original authors by request. Data from the National Genomic Research Library (NGRL) used in this research are available within the secure Genomics England Research Environment. Access to NGRL data is restricted to adhere to consent requirements and protect participant privacy. Data used in this research include participant phenotypic data, whole genome sequencing data and clinical data such as cancer diagnoses and mortality obtained from linked health records. Access to NGRL data is provided to approved researchers who are members of the Genomics England Research Network, subject to institutional access agreements and research project approval under participant-led governance. For more information on data access, visit: https://www.genomicsengland.co.uk/research.
